# Stratification of multiple sclerosis patients using unsupervised machine learning: a single-visit MRI-driven approach

**DOI:** 10.1007/s00330-022-08610-z

**Published:** 2022-03-14

**Authors:** Giuseppe Pontillo, Simone Penna, Sirio Cocozza, Mario Quarantelli, Michela Gravina, Roberta Lanzillo, Stefano Marrone, Teresa Costabile, Matilde Inglese, Vincenzo Brescia Morra, Daniele Riccio, Andrea Elefante, Maria Petracca, Carlo Sansone, Arturo Brunetti

**Affiliations:** 1 Department of Advanced Biomedical Sciences, University “Federico II”, Via Pansini 5, 80131 Naples, Italy; 2 Department of Electrical Engineering and Information Technology (DIETI), University “Federico II”, Naples, Italy; 3 Institute of Biostructure and Bioimaging, National Research Council, Naples, Italy; 4 Department of Neurosciences and Reproductive and Odontostomatological Sciences, University “Federico II”, Naples, Italy; 5 Multiple Sclerosis Centre, II Division of Neurology, Department of Clinical and Experimental Medicine, “Luigi Vanvitelli” University, Naples, Italy; 6 Department of Neuroscience, Rehabilitation, Ophthalmology, Genetics, Maternal and Child Health (DINOGMI), University of Genoa, Genoa, Italy; 7 Ospedale Policlinico San Martino IRCCS, Genoa, Italy

**Keywords:** Multiple sclerosis, Brain, Magnetic resonance imaging, Machine learning, Prognosis

## Abstract

**Objectives:**

To stratify patients with multiple sclerosis (pwMS) based on brain MRI-derived volumetric features using unsupervised machine learning.

**Methods:**

The 3-T brain MRIs of relapsing-remitting pwMS including 3D-T1w and FLAIR-T2w sequences were retrospectively collected, along with Expanded Disability Status Scale (EDSS) scores and long-term (10 ± 2 years) clinical outcomes (EDSS, cognition, and progressive course). From the MRIs, volumes of demyelinating lesions and 116 atlas-defined gray matter regions were automatically segmented and expressed as *z*-scores referenced to external populations. Following feature selection, baseline MRI-derived biomarkers entered the Subtype and Stage Inference (SuStaIn) algorithm, which estimates subgroups characterized by distinct patterns of biomarker evolution and stages within subgroups. The trained model was then applied to longitudinal MRIs. Stability of subtypes and stage change over time were assessed via Krippendorf’s *α* and multilevel linear regression models, respectively. The prognostic relevance of SuStaIn classification was assessed with ordinal/logistic regression analyses.

**Results:**

We selected 425 pwMS (35.9 ± 9.9 years; F/M: 301/124), corresponding to 1129 MRI scans, along with healthy controls (*N* = 148; 35.9 ± 13.0 years; F/M: 77/71) and external pwMS (*N* = 80; 40.4 ± 11.9 years; F/M: 56/24) as reference populations. Based on 11 biomarkers surviving feature selection, two subtypes were identified, designated as “deep gray matter (DGM)-first” subtype (*N* = 238) and “cortex-first” subtype (*N* = 187) according to the atrophy pattern. Subtypes were consistent over time (*α* = 0.806), with significant annual stage increase (*b* = 0.20; *p* < 0.001). EDSS was associated with stage and DGM-first subtype (*p* ≤ 0.02). Baseline stage predicted long-term disability, transition to progressive course, and cognitive impairment (*p* ≤ 0.03), with the latter also associated with DGM-first subtype (*p* = 0.005).

**Conclusions:**

Unsupervised learning modelling of brain MRI-derived volumetric features provides a biologically reliable and prognostically meaningful stratification of pwMS.

**Key Points:**

*• The unsupervised modelling of brain MRI-derived volumetric features can provide a single-visit stratification of multiple sclerosis patients.*

*• The so-obtained classification tends to be consistent over time and captures disease-related brain damage progression, supporting the biological reliability of the model.*

*• Baseline stratification predicts long-term clinical disability, cognition, and transition to secondary progressive course.*

**Supplementary Information:**

The online version contains supplementary material available at 10.1007/s00330-022-08610-z.

## Introduction

Brain MRI abnormalities in multiple sclerosis (MS) represent objective indicators of the patient’s biological status, reflecting pathogenetic mechanisms underlying disease evolution [1].

Although a massive body of evidence regarding the biological and clinical relevance of MRI biomarkers has been provided through the years by large-N research studies, their implementation in the single-subject setting and therefore in clinical practice remains challenging [2]. Actually, MRI biomarkers exhibit high variance, resulting from both non-disease-related confounders (e.g., age, sex, other coexisting CNS physiologic and pathologic conditions) and disease-related phenotypic and temporal heterogeneity, thus hampering the definition of absolute cut points and limiting their utility for effective patient stratification.

Over the years, technical advances and the emergence of imaging guidelines [3, 4] have led to the widespread availability of high-quality clinical MRI scans, including sequences with isotropic voxel resolution suitable for volumetric quantifications [5]. Unfortunately, this goldmine of information remains largely unexploited due to the complexity of meaningfully modelling high-dimensional dataset and the frequent lack of associated data reliably reflecting the patients’ clinical status.

Unsupervised machine learning (ML) techniques modelling disease progression based solely on objective biomarker changes, without reliance on a priori clinical information or explicit biomarker thresholds, represent a valuable approach to overcome these issues [6]. Recently, such methods have been applied to primary neurodegenerative disorders of the central nervous system [6–8] and showed promising results when translated into the MS scenario with the aim to characterize the disease-specific sequence of clinical and MRI changes [9, 10] or to provide an MRI-driven definition of disease phenotypes [11].

Based on these premises, we applied a recently developed algorithm called Subtype and Staging Inference (SuStaIn), which identifies data-driven subtypes characterized by distinct trajectories of biomarker abnormality accumulation, to clinical MRI scans of a large single-center cohort of relapsing-remitting MS (RRMS) patients. We aimed to demonstrate that, based on a fine-grained volumetric mapping of different brain areas and MS lesions obtained from cross-sectional MRI visits, such approach would provide an accurate patient stratification which is both biologically reliable and prognostically meaningful in the light of longitudinal MRIs and long-term (10-year) motor and cognitive evaluations.

## Materials and methods

### Participants

**Fig. 1:**
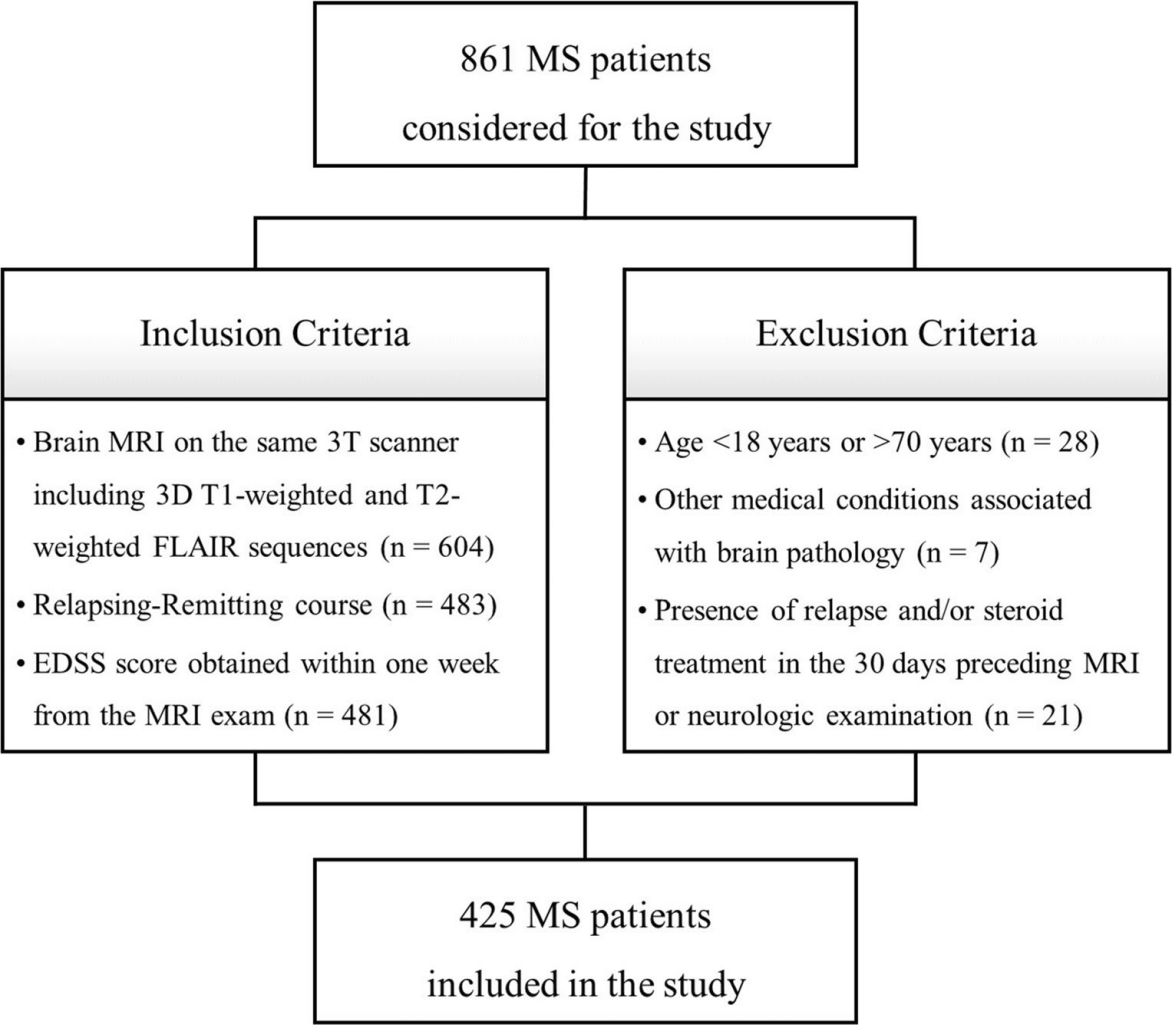
Flowchart showing inclusion and exclusion criteria. Overall, 861 MS patients were considered for this study. After application of the inclusion and exclusion criteria, a total of 425 patients were selected, corresponding to 1129 MRI scans

In this monocentric retrospective study, brain MRI studies of patients with an MS diagnosis revised according to the 2010 McDonald criteria [12] and a relapsing-remitting (RR) course [13] were screened for eligibility from the radiological and clinical research databases of the MS center of the University of Naples “Federico II,” containing data collected starting from October 2006. Inclusion and exclusion criteria are shown in Fig. 1.

Brain MRI scans of healthy controls (HC) from the same databases and an external population of RRMS patients from the University of Genoa were also selected to develop norms for *z*-score calculation and select MRI features.

The study was conducted in compliance with the ethical standards and approved by the Ethics Committee “Carlo Romano” of the host institution.

### Clinical evaluation

For all patients, clinical disability within 1 week from MRI was estimated using the Expanded Disability Status Scale (EDSS). Patients for whom a long-term clinical and neuropsychological evaluation was available were classified at follow-up (10 ± 2 years from baseline MRI) according to the following: (i) motor disability, ranging from 0 to 3 according to ambulation benchmarks corresponding to EDSS scores < 4.0, ≥ 4.0 and < 6.0, ≥ 6.0 and < 7.0, ≥ 7.0 [14]; (ii) cognitive disability, ranging from 0 to 3 and corresponding to the number of impaired (below 1.5 SD age-, sex- and education-corrected normative values in the healthy population [15]) tests at the Brief International Cognitive Assessment of Multiple Sclerosis (BICAMS) battery [16]; (iii) transition to secondary progressive course [13].

### MRI data acquisition and processing

Exams were acquired on the same 3-T scanner (Magnetom Trio, Siemens Healthineers) and included a 3D T1-weighted sequence (≤ 1-mm isotropic voxel size) for volumetric analyses and a T2-weighted FLAIR sequence for the quantification of total demyelinating lesion volume (TLV). Sequence parameters and image processing steps are detailed in the Supplemental Material. Briefly, for all participants, demyelinating lesions were automatically segmented, visually checked, and where needed manually adjusted on FLAIR images to compute TLV, while T1-weighted volumes were used for an atlas-based parcellation of gray matter (GM) into 116 regions defined by the Automated Anatomical Labeling (AAL) atlas [17].

### Statistical analysis

**Fig. 2:**
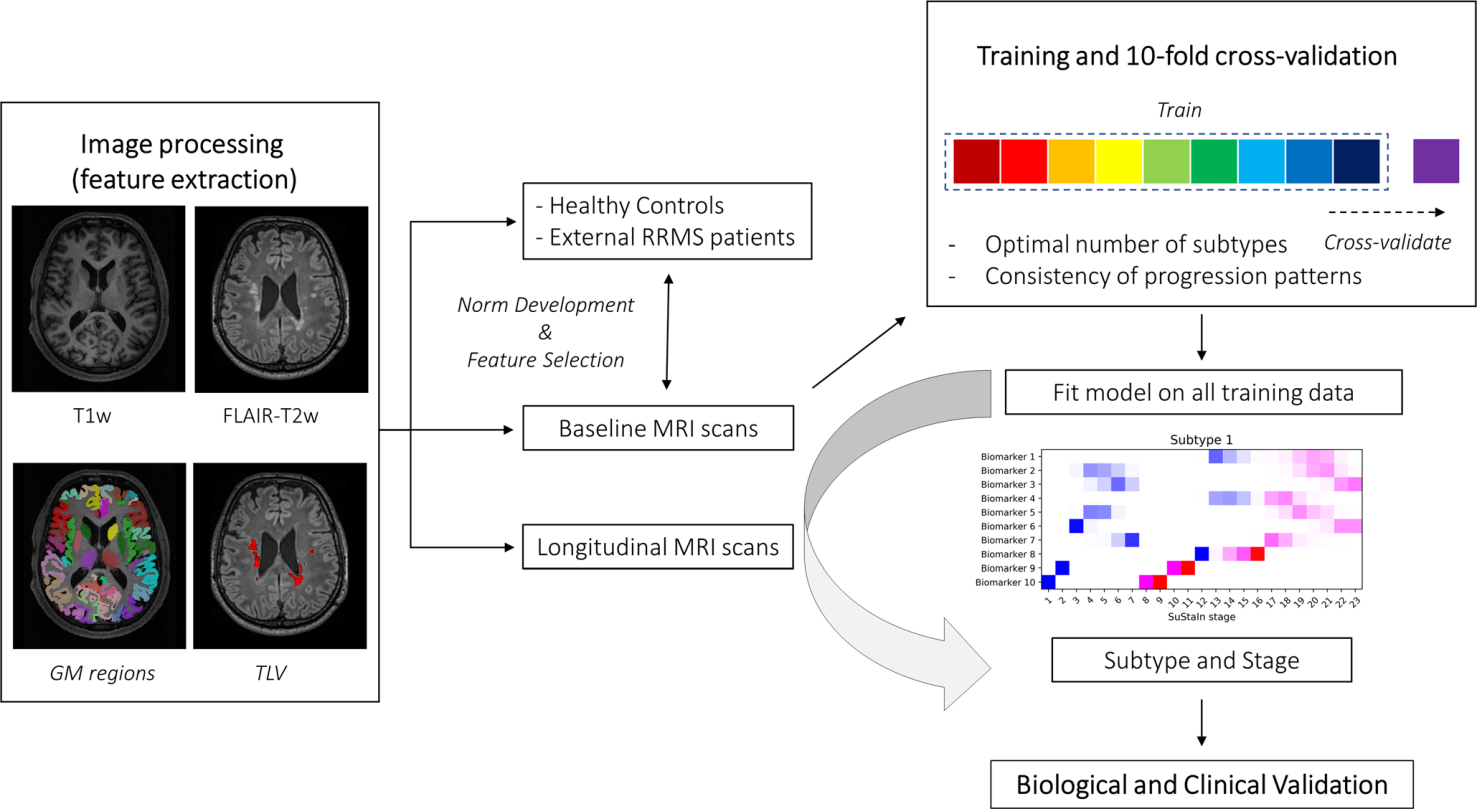
Workflow illustrating the main data processing and analysis steps. Volumes of demyelinating lesions and 116 atlas-defined gray matter regions were automatically segmented based on FLAIR-T2w and T1-w images, respectively. Then, the corresponding volumes were expressed as *z*-scores with reference to external populations of patients and healthy controls that were also used to select the most altered MRI-derived volumes. Following feature selection, baseline MRI biomarkers entered the Subtype and Stage Inference (SuStaIn) algorithm, using 10-fold cross-validation to determine the optimal number of subtypes and the consistency of progression patterns. Models of up to a maximum of 4 subtypes with *z*-scores of 1, 2, or 3 for each biomarker were tested (excluding *z*-score events reached by fewer than 5% of the subjects), corresponding to interpretable levels of mild, moderate, and severe abnormality (color coded from blue to red). The trained model was then fit on all training data and applied to longitudinal MRIs. Finally, the biological reliability and clinical relevance of the SuStaIn classification were assessed in the light of longitudinal MRI scans and clinical outcomes

A flowchart summarizing data processing and analysis steps is depicted in Fig. 2.

#### SuStaIn modelling

SuStaIn is an unsupervised machine learning algorithm combining ideas from clustering and event-based modelling, which describes disease progression as the linear evolution of biomarkers along discrete levels of cumulative alteration, defined in terms of deviation from a reference norm (*z*-scores) [8]. It simultaneously estimates subgroups characterized by distinct patterns of biomarker evolution and the corresponding trajectories, providing a probabilistic assignment of each subject to a specific subtype and stage within a subtype [8]. Methodological aspects of the SuStaIn algorithm are covered in Young et al [8], while the details of the current analysis are provided in the Supplemental Material.

Briefly, MRI-derived GM and lesion volumes were expressed as *z*-scores with reference to the HC group and the external RRMS population, respectively, with signs of the *z*-scores flipped when appropriate so that higher values always represented disease worsening. Baseline MRI scans were used as the training set, while longitudinal visits were reserved for the biological and clinical validation of the initial classification [8].

Only variables associated with a moderate to large (Cohen’s *f* > 0.25) difference between MS patients and HC were selected and entered the SuStaIn algorithm. Models were evaluated using 10-fold cross-validation (CV) in the training cohort to estimate the optimal number of subtypes and the consistency of the subtype progression patterns: the number of subtypes maximizing the average out-of-sample log-likelihood across CV folds was preferred; the similarity of each subtype progression pattern across CV folds (CVS) was measured using the Bhattacharyya coefficient [8]. Finally, the resulting model was fitted on all subjects of the training cohort and applied to unseen longitudinal MRI scans to assign a probable subtype and stage to each MRI visit.

#### Testing the biological reliability and clinical relevance of SuStaIn classification

The stability of the SuStaIn subtypes over time was expressed with Krippendorf’s *α* [18]. To assess the rate of change in disease stage, we fit a multilevel linear regression model in which the SuStaIn stage was the dependent variable and follow-up time (nested within subjects) the predictor, with intercepts and slopes allowed to vary across subjects (random effects). The possible effect of baseline subtype and stage on the slope of longitudinal stage change was assessed by separately adding them (and the corresponding interactions with follow-up time) to the model and testing the significance of interaction terms. Similar models were set up for individual MRI-derived biomarkers.

The clinical relevance of the SuStaIn classification was assessed in relation to both baseline EDSS and long-term clinical outcomes with ordinal/logistic regression (as appropriate) analyses, in which baseline subtype and stage and their interaction, age, and sex were the independent variables. Follow-up time, baseline EDSS, and disease-modifying therapy were included as additional covariates for longitudinal analyses.

Statistical analyses were carried out using the Statistical Package for Social Science (SPSSv25.0, IBM corp.).

## Results

### Participants

Four hundred and twenty-five RRMS patients (baseline age: 35.9 ± 9.9 years; F/M: 301/124) were selected, corresponding to a total of 1129 MRI visits (2.7 MRI visits per patient, on average; range: 0–9), and a mean follow-up (FU) time of 2.1 years.

MRI scans of 148 HC (age: 35.9 ± 13.0 years; F/M: 77/71) were also selected, along with those of an external population of 80 MS patients (age: 40.4 ± 11.9 years; F/M: 56/24).

Demographic and clinical characteristics of the studied population are reported in Table 1.

**Table 1:** Demographic, clinical, and MRI characteristics of the studied population

	MS	HC	MS (external site)
Number of subjects	425	148	80
Number of MRI scans	1129	148	80
Age (y)	35.9 ± 9.9	35.9 ± 13.0	40.4 ± 11.9
Female Sex*	301 (70.8)	77 (52.0)	56 (70.0)
DD (y)	12.7 ± 8.3	-	10.3 ± 7.4
EDSS**	2.5 (2.0 - 3.5)	-	2.0 (1.5 - 3.0)
TLV (mL)	10.1 ± 13.4	-	3.4 ± 5.3
WBV (mL)	1328.8 ± 127.9	1385.1 ± 147.4	1370.4 ± 153.3

*Data are the number of subjects, with percentages in parentheses.

**Data are medians, with interquartile ranges in parentheses.

*MS*, multiple sclerosis; *HC*, healthy controls; *DD*, disease duration; *EDSS*, Expanded Disability Status Scale; *TLV*, total lesion volume; *WBV*, whole brain volume

Long-term clinical outcomes were available for 178 patients (level of motor disability: 0 = 121, 1 = 35, 2 = 16, 3 = 6; level of cognitive disability: 0 = 81, 1 = 42, 2 = 24, 3 = 31; transition to secondary progressive course: 29 subjects).

### SuStaIn model

**Fig. 3:**
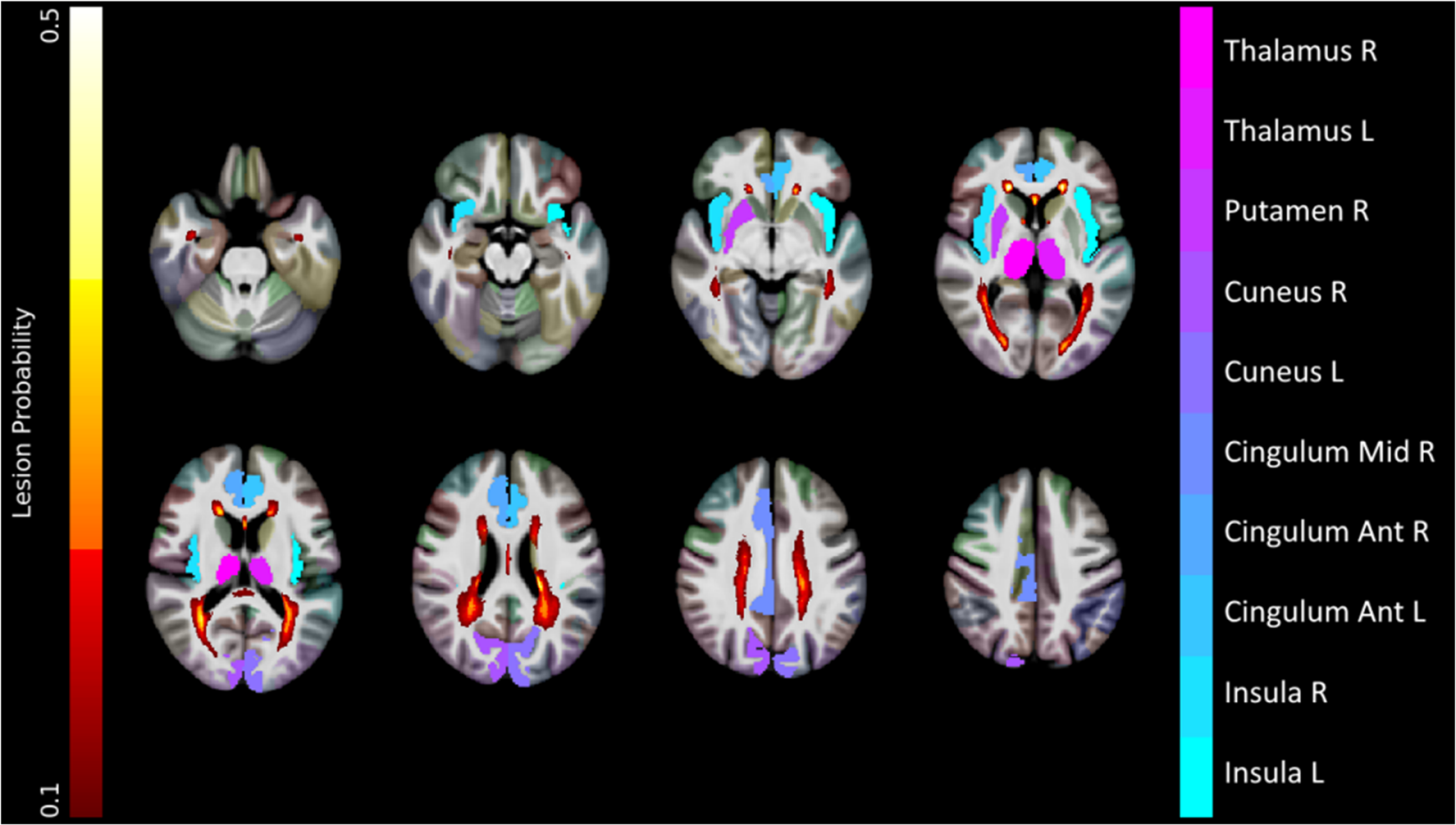
Results of the feature selection procedure. Gray matter regions whose volume survived the feature selection procedure (i.e., associated with a moderate to large effect size at the comparison with healthy controls) are presented, along with a lesion probability map (obtained by summing all the binary lesion masks and dividing by the number of patients, thresholded at 10% probability), all superimposed on axial slices of the average T1w volume in the standard space. Images are in radiological orientation

The volumes of 10 GM regions, including the bilateral anterior cingulate cortices, the right middle cingulate cortex, the bilateral insulae and cunei, the right putamen, and the bilateral thalami, were associated with a moderate to large difference compared with the HC group and were thus fed into the SuStaIn algorithm along with TLV, for a total of 11 biomarkers (Fig. 3, Supplementary Table 1).

**Fig. 4:**
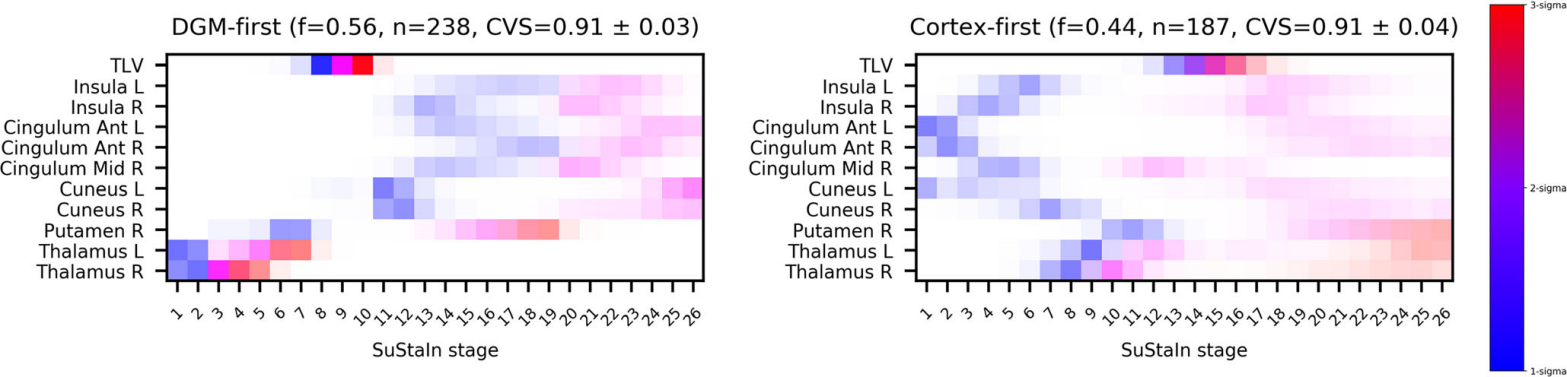
Positional variance diagrams for the two MRI-driven subtypes. Each entry describes the probability for each biomarker of reaching the color-coded *z*-score at each SuStaIn stage. The colors represent the degree of abnormality based on the *z*-score level (*blue* = mild, *z*-score of 1; *violet* = moderate, *z*-score of 2; *red* = severe, *z*-score of 3), while the color shade reflects the uncertainty associated with the corresponding biomarker event. CVS, cross-validation similarity; TLV, total lesion volume

The two-subtype model yielded the highest average log-likelihood across CV folds (Supplementary Figure 1) and was therefore chosen as the best fitting model for subsequent analyses. When looking at the trajectories of brain damage progression in each subtype, we designated them as follows: (1) the deep gray matter (DGM)-first subtype (56% of subjects, *n* = 238), characterized by the initial volume loss of subcortical gray matter structures followed by lesion accrual and cortical atrophy; and (2) the cortex-first subtype (44% of subjects, *n* = 187), characterized by cortical volume loss preceding DGM atrophy and lesion accumulation (Fig. 4).

Both progression patterns demonstrated high stability across CV folds, with CVS of 0.91 ± 0.03 and 0.91 ± 0.04 for the DGM-first and cortex-first subtypes, respectively (Supplementary Figure 2).

Patients assigned to the two subtypes had comparable age, sex, and whole brain volume (WBV), while the DGM-first subtype was associated with longer DD (*p* < 0.001) and higher baseline EDSS score (*p* = 0.004), SuStaIn stage (*p* = 0.01), and TLV (*p* < 0.001) (Table 2).

**Table 2:** Demographic, clinical and MRI characteristics of the MRI-driven subtypes

	DGM-first (56%, *n* = 238)	Cortex-first (44%, *n* = 187)	*p*-value***
Age (y)	35.9 ± 10.1	35.9 ± 9.5	0.98
Female Sex*	160 (67.2)	141 (75.4)	0.36
DD (y)	9.4 ± 7.8	6.5 ± 6.1	**< 0.001**
EDSS**	2.5 (2.0-3.5)	2.5 (2.0-3.0)	**0.004**
SuStaIn stage	4 (1-12)	4 (1-8)	**0.01**
TLV (mL)	14.0 ± 15.1	5.5 ± 8.9	**< 0.001**
WBV (mL)	1325.3 ± 126.8	1333.0 ± 129.5	0.65

Unless otherwise indicated, data are expressed as mean ± standard deviation. Between-group differences were tested with either Student *t* (age and DD), Pearson Chi-square (sex), Kruskal-Wallis (EDSS and SuStaIn stage), or age-, sex-, and TIV-corrected ANCOVA (TLV and WBV) tests.

*Data are the number of subjects, with percentages in parentheses.

**Data are medians, with interquartile ranges in parentheses.

***Significant between-group differences are reported in bold.

*DGM*, deep gray matter; *DD*, disease duration; *EDSS*, Expanded Disability Status Scale; *TLV*, total lesion volume; *WBV*, whole brain volume.

### Biological reliability and clinical relevance

Disease subtypes tended to be consistent over time (Krippendorf’s *α* = 0.806; CI = 0.752, 0.821), with subtype stability increasing as the probability threshold for the baseline subtype assignment was raised at 95% (177 subjects; *α* = 0.990; CI = 0.973, 0.998) or 99% (114 subjects; *α* = 0.990; CI = 0.973, 0.998).

In patients who retained the initial subtype, there was a significant annual increase in disease stage (*b* = 0.20; SE = 0.05; CI = 0.09, 0.30; *p* < 0.001), supporting the biological reliability of SuStaIn’s staging, with no significant between-subtype difference (interaction term subtype*follow-up time: *b* = −0.08; SE = 0.11; CI = −0.29, 0.13; *p* = 0.48). A significant moderation effect of baseline stage on the relationship between follow-up time and disease stage was observed (interaction term baseline stage*follow-up time: *b* = −0.05; SE = 0.01; CI = −0.08, −0.02; *p* = 0.001), corresponding to slopes getting flatter as the baseline stage increased and probably reflecting a plateau effect.

When looking at individual MRI-derived biomarkers, all the GM volumes significantly decreased over time (*p* ≤ 0.03) (Supplementary Table 2), with significant between-group differences for the left thalamus, corresponding to greater longitudinal atrophy rates in the DGM-first subtype (interaction term subtype*follow-up time: *b* = 0.05; SE = 0.01; CI = 0.02, 0.07; *p* = 0.001), and significant plateau effects (the higher the baseline stage, the flatter the slope of longitudinal changes) for the right thalamus (interaction term subtype*follow-up time: *b* = 0.007; SE = 0.003; CI = 0.002, 0.013; *p* = 0.006) and the right anterior cingulate cortex (interaction term subtype*follow-up time: *b* = 0.002; SE = 0.001; CI = 0.001, 0.003; *p* = 0.002).

As for the relationship with clinical outcomes, baseline EDSS score was positively related with both SuStaIn stage (*b* = 0.042; *p* < 0.001) and the DGM-first subtype (*b* = −0.280; *p* = 0.02), with baseline stage that also predicted long-term disability (*b* = 0.030; *p* = 0.007) and transition to SP course (*b* = 0.079; *p* = 0.03). Long-term cognitive impairment was associated with higher baseline stages (*b* = 0.048; *p* < 0.001), the DGM-first subtype (*b* = −0.442; *p* = 0.005), and their interaction (*b* = −0.080; *p* = 0.002) (Table 3).

**Table 3:** Results of the regression analyses for the prediction of clinical outcomes. For both ordinal (baseline and long-term EDSS and long-term BICAMS) and logistic (transition to SP course) regression analyses, the overall fit (*R*^2^ and *F*-statistic for ordinal, Nagelkerke *R*^2^ and -2LL for logistic regression) and associated significance level of the model are presented, along with the estimated parameters (and corresponding 5000-resamples bootstrap 95% CI and SE) and associated test statistic (*t* for ordinal, *z* for logistic regression) and significance level of both the intercept and individual predictors

	Model			Predictor			
	*R*^2^/Nagelkerke *R*^2^	*F*/-2LL	*p*	*b* (95% CI)	SE	*t*/*z*	*p*
Baseline EDSS	0.256	27.245	< 0.001				
Constant				1.597 (1.243, 1.952)	0.180	57.333	**< 0.001**
SuStaIn subtype				−0.280 (−0.460, −0.100)	0.092	−3.056	**0.02**
SuStaIn stage				0.042 (0.027, 0.058)	0.008	5.368	**< 0.001**
SuStaIn subtype x stage				−0.012 (−0.042, 0.018)	0.015	−0.703	0.43
Age				0.033 (0.023, 0.042)	0.005	6.639	**< 0.001**
Sex				−0.120 (−0.305, 0.066)	0.095	−1.263	0.21
Long-term EDSS	0.291	6.253	< 0.001				
Constant				0.654 (−0.330, 1.637)	0.498	1.311	0.19
SuStain subtype				−0.059 (−0.287, 0.170)	0.116	−0.507	0.61
SuStaIn stage				0.030 (0.008, 0.052)	0.011	2.726	**0.007**
SuStaIn subtype x stage				−0.016 (−0.059, 0.028)	0.022	−0.707	0.48
Age				0.015 (0.002, 0.028)	0.006	2.353	**0.02**
Sex				0.004 (−0.248, 0.256)	0.128	0.029	0.98
FU time				−0.112 (−0.207, −0.016)	0.049	−2.296	**0.02**
DMT**				0.280 (0.102, 0.457)	0.090	3.113	**0.002**
Long-term BICAMS	0.287	13.492	< 0.001				
Constant				−0.269 (−1.798, 1.259)	0.774	−0.248	0.73
SuStain subtype				−0.442 (−0.751, −0.133)	0.157	−2.824	**0.005**
SuStaIn stage				0.048 (0.024, 0.072)	0.012	3.946	**< 0.001**
SuStaIn subtype x stage				−0.080 (−0.130, −0.030)	0.025	−3.160	**0.002**
Age				0.001 (−0.017, 0.020)	0.009	0.120	0.90
Sex				0.196 (−0.140, 0.532)	0.170	1.151	0.25
FU time				0.110 (−0.040, 0.261)	0.076	1.450	0.15
DMT**				0.093 (−0.160, 0.346)	0.128	0.726	0.47
Long-term SP course*	0.299	121.230	< 0.001				
Constant				−2.973 (−7.442, −1.496)	2.280	−1.304	0.19
SuStain subtype				0.422 (−0.556, 1.399)	0.499	0.846	0.40
SuStaIn stage				0.079 (0.009, 0.149)	0.036	2.204	**0.03**
SuStaIn subtype x stage				0.044 (−0.103, 0.191)	0.075	0.586	0.56
Age				0.095 (0.035, 0.155)	0.031	3.091	**0.002**
Sex				−0.926 (−2.116, 0.263)	0.607	−1.526	0.13
FU time				−0.301 (−0.694, 0.092)	0.200	−1.503	0.13
DMT**				0.571 (0.006, 1.135)	0.288	1.980	**0.048**

For all analyses, the DGM-first subtype was coded as 0 and the Cortex-first as 1.

Significant values are reported in bold

*Long-term course was coded as follows: SP course = 1, RR course = 0.

**DMT was coded as follows: no therapy = 0 (13 patients, 7.3%), interferon = 1 (140 patients, 78.7%), glatiramer acetate = 2 (5 patients, 2.8%), natalizumab = 3 (20 patients, 11.2%).

*SP*, secondary progressive; *RR*, relapsing remitting; *LL*, log-likelihood; *CI*, confidence interval; *SE*, standard error; *FU*, follow-up; *DMT*, disease-modifying therapy.

## Discussion

The ambition towards personalized medicine has stimulated increasing efforts to disentangle the inter-subject variability of neurological disorders, integrating information from different biomarkers to identify distinct underlying biological drivers (i.e., biotypes), up to the level of individual patients [19]. In this work, we obtained a biologically consistent and prognostically relevant stratification of RRMS patients based on the unsupervised modeling of brain volumetric features derived from cross-sectional MRI visits.

Using the SuStaIn algorithm, two distinct MRI-driven subtypes were identified, with a latent pattern in which early DGM atrophy and T2 lesion accumulation precede cortical atrophy separated from one in which cortical volume loss precedes DGM atrophy and lesion accrual. These results are essentially in line with the recent work by Eshaghi et al [11], with slight dissimilarities most probably due to the different choices of input features. Indeed, the apparent discrepancy in terms of the number of subtypes is most likely explained by the lack of MRI-derived measures of normal appearing white matter damage in our study, which limited the sensitivity to capture the phenotypic heterogeneity associated with extra-lesional microstructural injury.

On the other hand, the application of a more fine-grained brain parcellation scheme led to a more anatomically precise modelling of GM atrophy, highlighting regions most prominently involved in MS such as the thalami and anterior cingulate, insular, and visual cortices [9]. Interestingly, the fact that distinct disease subtypes remain distinguishable based on the patients’ MRIs even within a relatively clinically homogeneous population confirms the scarce correspondence between clinical and MRI-driven phenotyping, with the latter more closely reflecting disease-related pathogenic mechanisms [11].

Indeed, while patients assigned to the two subtypes did not significantly differ in terms of age, sex, or WBV, the DGM-first subtype was associated with higher DD, stage, and TLV, consistent with the idea of distinct pathogenic mechanisms underpinning cortical and DGM atrophy [20–22]. In particular, based on the closer association with TLV, subcortical GM might be more sensitive to the secondary effects of focal demyelination through anterograde/retrograde degeneration, with a prominent role of primary GM neuroinflammation and neurodegeneration in determining cortical atrophy [21–23]. Also, the longer DD suggest an earlier diagnosis in patients of the DGM-first subtype, possibly reflecting a shorter prodromal phase [11, 24].

The biological reliability of the MRI-driven classification was further confirmed by the analysis of longitudinal MRI scans, with high subtype stability and significant stage increase over time, reflecting actual temporal progression of brain damage along the estimated paths. Also relevant in terms of biological consistency, moderation analyses suggested plateau effects in the longitudinal trajectories of SuStaIn stage and individual biomarkers (i.e., right thalamus and anterior cingulate cortex atrophy), in line with known temporal patterns of MS-related brain atrophy [25, 26], with steeper thalamic shrinkage rates in the DGM-first subtype.

When assessing the clinical relevance of the SuStaIn classification, higher baseline EDSS scores were independently associated with both higher stages, corresponding to more pronounced brain structural damage, and the DGM-first subtype, a finding consistent with prior evidence pointing at the prominent role of subcortical GM (thalamic, in particular) atrophy in driving disability [27, 28].

As for the prognostic meaning of the MRI-driven stratification, patients in a more advanced position along the damage progression trajectory were more likely to enter the clinically progressive phase in the long term, as well as to suffer greater degrees of motor and cognitive disability, with more severe cognitive impairment also independently associated with the DGM-first subtype. These findings further corroborate the idea that, although cross-sectional in nature, the baseline MRI-driven classification encodes relevant information about future disease evolution, also substantiating the role of subcortical GM atrophy as a relevant anatomical correlate of cognitive disability in MS [29, 30].

Overall, the proposed approach provides insights into MS-related disease mechanisms, confirming and expanding the existing knowledge on MS physiopathology. But even more interestingly, it condenses this complex information at the patient level in simple and intuitive measures which are easily obtainable from single-visit conventional MRI scans and correlate with clinical measures of disease severity and progression. Contextualizing the information contained in individual brain MRIs in the frame of disease patterns estimated in a reference population of MS patients, such stratification holds potential for effectively linking MS research to the single-subject setting, with relevant implications for both clinical trials and routine practice.

Our work is not without limitations. While the monocentric nature of the study reduces the data heterogeneity related to scanner/center effects, it also limits the model generalizability, prompting larger studies on multicentric datasets. Furthermore, increasing the sample size would also allow for a higher dimensional (and more accurate) representation of MS pathology, possibly including additional biomarkers from spinal cord imaging or from other advanced MRI techniques encoding relevant information about the brain microstructure (e.g., diffusion MRI, quantitative MRI) [20, 31] or function (e.g., functional MRI) [32].

In conclusion, through the unsupervised modelling of volumetric features derived from brain MRI scans, we obtained a biologically reliable and prognostically meaningful single-visit classification of MS patients, potentially offering a powerful tool for subjects’ stratification in both trial design and clinical practice.

## Supplementary Information

ESM 1 (DOCX 711 kb)

## Abbreviations

DD: Disease duration
DGM: Deep gray matter
DMT: Disease-modifying therapy
EDSS: Expanded Disability Status Scale
GM: Gray matter
HC: Healthy controls
MS: Multiple sclerosis
RR: Relapsing remitting
TLV: Total lesion volume
WBV: Whole brain volume

## Declarations

### Guarantor

The scientific guarantor of this publication is Giuseppe Pontillo, MD.

### Statistics and biometry

One of the authors has significant statistical expertise.

### Methodology

• retrospective

• observational

• performed at one institution
