# Supplementary material for: Stratification of multiple sclerosis patients using unsupervised machine learning: a single-visit MRI-driven approach
Source: Eur Radiol. 2022 Mar 14;32(8):5382–91. doi: 10.1007/s00330-022-08610-z (PMC9279232; doi:10.1007/s00330-022-08610-z)
Supplement: Supplementary file 1 — (DOCX 711 kb) [file 330_2022_8610_MOESM1_ESM.docx]

**Stratification of Multiple Sclerosis patients using unsupervised machine learning: a single-visit MRI-driven approach**

**Materials and Methods**

***MRI data acquisition***

Exams were acquired on the same 3T scanner (Magnetom Trio, Siemens Healthineers), equipped with an 8-channel head coil, with the following protocols:

*Protocol 1 (887 timepoints)*

- 3D T1-weighted Magnetization Prepared Rapid Acquisition Gradient Echo sequence (MPRAGE; TR=1900 ms; TE=3.4 ms; TI=900 ms; Flip Angle=9°; resolution=1x1x1 mm^3^; 160 axial slices)

- 2D T2-weighted Fluid Attenuated Inversion Recovery sequence (FLAIR; TR=8500 ms; TE=106 ms; TI=2500 ms; Flip Angle=150°; voxel size=0.9x0.9x4 mm^3^; 25 axial slices)

*Protocol 2 (111 timepoints)*

- 3D T1-weighted Magnetization Prepared Rapid Acquisition Gradient Echo sequence (MPRAGE; TR=2500 ms; TE=2.8 ms; TI=900 ms; Flip Angle=9°; resolution=1x1x1 mm^3^; 176 sagittal slices)

- 3D T2-weighted Fluid Attenuated Inversion Recovery sequence (FLAIR; TR=6000 ms; TE=396 ms; TI=2200 ms; Flip Angle=120°; voxel size=1x1x1 mm^3^; 160 sagittal slices)

*Protocol 3 (75 timepoints)*

- 3D T1-weighted Magnetization Prepared Rapid Acquisition Gradient Echo sequence (MPRAGE; TR=1900 ms; TE=3.4 ms; TI=900 ms; Flip Angle=9°; resolution=1x1x1 mm^3^; 160 axial slices)

- 3D T2-weighted Fluid Attenuated Inversion Recovery sequence (FLAIR; TR=6000 ms; TE=404 ms; TI=2200 ms; Flip Angle=120°; voxel size=1x1x1 mm^3^; 160 sagittal slices)

*Protocol 4 (56 timepoints)*

- 3D T1-weighted Magnetization Prepared Rapid Acquisition Gradient Echo sequence (MPRAGE; TR=3000 ms; TE=2.4 ms; TI=1000 ms; Flip Angle=7°; resolution=0.8x0.8x0.8 mm^3^; 224 sagittal slices)

- 3D T2-weighted Fluid Attenuated Inversion Recovery sequence (FLAIR; TR=6000 ms; TE=404 ms; TI=2200 ms; Flip Angle=120°; voxel size=1x1x1 mm^3^; 160 sagittal slices)

***Image processing***

For all analyses, different MRI visits were considered as separate instances using a cross-sectional image processing pipeline[1].

To take into account for possible differences in terms of voxel-size and orientation, T1-weighted and FLAIR images were automatically reoriented and resampled to 1mm isotropic resolution by rigidly aligning them to corresponding templates in the MNI space using the Statistical Parametric Mapping software package (SPM12, http://www.fil.ion.ucl.ac.uk/spm).

For all participants, demyelinating lesions were automatically segmented on FLAIR images using the lesion prediction algorithm[2] implemented in the Lesion Segmentation Tool (LST) toolbox v3.0.0 (www.statistical-modelling.de/lst.html) for SPM. Lesion probability maps were then used to fill lesions in T1-weighted images for subsequent processing steps via LST’s default lesion filling procedure, and binarized (thresholding at 0.5 probability) to compute TLV. Lesion masks resulting from the automatic segmentation procedure were visually inspected by a neuroradiologist with more than five years of experience in the field of neuroimaging (G.P.), and manually adjusted when necessary.

Filled T1-weighted volumes were processed via the segmentation/normalization pipeline implemented in the Computational Anatomy Toolbox (CAT12.6, http://www.neuro.uni-jena.de/cat) for SPM, using the default settings (http://dbm.neuro.uni-jena.de/cat12/CAT12-Manual.pdf), including spatial normalization to CAT12 built-in DARTEL template and use of the inverse spatial transformation to obtain an atlas-based parcellation in the individual subject’s space of gray matter (GM) volume into 116 regions defined according to the Automated Anatomical Labeling (AAL) atlas[3]. Whole brain volume (WBV) was also computed as the sum of GM and WM binary tissue maps and total intracranial volume (TIV) was estimated using CAT12 standard procedure.

***SuStaIn modelling***

Initially, MRI-derived volumes were expressed as z-scores with reference to the HC group, adjusting for the effect of age, sex and TIV. For each metric, the linear relationship with these variables was modeled in the HC group and used to compute standardized residuals (z-scores) in all subjects. To obtain comparable (and more meaningful) z-score levels, TLV was referenced to values obtained in the external RRMS population. When appropriate (for tissue volumes) signs of the z-scores were flipped so that higher values and estimated stages would represent disease worsening.

Baseline MRI scans were used as the training set, while longitudinal visits were reserved for the biological and clinical validation of the initial classification[4].

To reduce the dimensionality of the models, variables which are more likely to be affected in MS were selected. To achieve this “biologically informed” feature selection, the difference between HC and MS patients at their baseline visit was tested in ANCOVA analyses correcting for age, sex and TIV and only those variables associated with a moderate to large effect size (Cohen’s *f* > 0.25) were retained. The resulting features were entered into the SuStaIn algorithm, testing models of up to a maximum of 4 subtypes with z-scores of 1, 2 or 3 for each biomarker, corresponding to interpretable levels of mild, moderate and severe abnormality. Z-score events reached by fewer than 5% of the subjects were excluded. The maximum z-score, which is reached at the final stage of the progression, was set by rounding up the 95% percentile of each biomarker to the nearest integer, with a plateau of 5[4]. Models were evaluated using 10-fold cross-validation (CV) in the training cohort to estimate the optimal number of subtypes and the consistency of the subtype progression patterns. The model with the number of subtypes that maximized the out-of-sample log-likelihood across CV folds was preferred, while the similarity of each subtype progression pattern across CV folds (CVS) was measured using the Bhattacharyya coefficient (ranging from 0, no agreement, to 1, perfect agreement), averaged across biomarker events and pairs of CV folds[4]. The resulting model was then fitted on all subjects of the training cohort and applied to unseen longitudinal MRI scans in order to assign a probable subtype, and stage within the subtype, to each MRI visit.

**Supplementary Tables**

**Supplementary Table 1. Results of the feature selection procedure.** For GM volumes surviving the feature selection procedure, effect sizes (Cohen’s *f*) of the difference between MS patients’ baseline visits and HCs (adjusted for age, sex and TIV) are given, along with corresponding 95% confidence intervals. All comparisons were statistically significant (*p*<0.0001).

| **GM volumes** | **Cohen’s *f* (95% CI)** |
| --- | --- |
| *Left Insula* | 0.26 (0.19, 0.33) |
| *Right Insula* | 0.30 (0.22, 0.37) |
| *Left Anterior Cingulate Cortex* | 0.27 (0.20, 0.34) |
| *Right Anterior Cingulate Cortex* | 0.26 (0.19, 0.33) |
| *Right Middle Cingulate Cortex* | 0.26 (0.19, 0.33) |
| *Left Cuneus* | 0.31 (0.24, 0.38) |
| *Right Cuneus* | 0.27 (0.20, 0.34) |
| *Right Putamen* | 0.26 (0.19, 0.33) |
| *Left Thalamus* | 0.40 (0.33, 0.47) |
| *Right Thalamus* | 0.42 (0.35, 0.49) |

GM=gray matter; CI=confidence interval.

**Supplementary Table 2. Annualized changes of individual MRI biomarkers.** Parameter estimates of the fixed effects of follow-up time for individual MRI biomarkers (expressed as z-scores) are presented, along with the corresponding standard errors, 95% confidence intervals and significance levels.

| **GM volumes (z-scores)** | ***b* (95% CI)** | ***SE*** | ***p*** |
| --- | --- | --- | --- |
| *Left Insula* | -0.032 (0.013, 0.050) | 0.009 | 0.001 |
| *Right Insula* | -0.021 (0.005, 0.036) | 0.008 | 0.01 |
| *Left Anterior Cingulate Cortex* | -0.016 (0.003, 0.028) | 0.006 | 0.01 |
| *Right Anterior Cingulate Cortex* | -0.012 (0.003, 0.027) | 0.006 | 0.02 |
| *Right Middle Cingulate Cortex* | -0.040 (0.022, 0.057) | 0.009 | <0.001 |
| *Left Cuneus* | -0.009 (0.001, 0.017) | 0.004 | 0.03 |
| *Right Cuneus* | -0.010 (0.003, 0.017) | 0.004 | 0.008 |
| *Right Putamen* | -0.064 (0.044, 0.085) | 0.010 | <0.001 |
| *Left Thalamus* | -0.070 (0.056, 0.084) | 0.007 | <0.001 |
| *Right Thalamus* | -0.068 (0.052, 0.084) | 0.008 | <0.001 |

GM=gray matter; CI=confidence interval; SE=standard error.

**Supplementary Figures**

**Supplementary Figure 1. Results of the 10-fold cross-validation.** For models with different number of subtypes, values of log-likelihood (LL) on the test data for each cross-validation fold are presented (*left panel*), along with the corresponding average cross-validation information criterion (CVIC, defined as -2*LL) (*right panel*)[4].


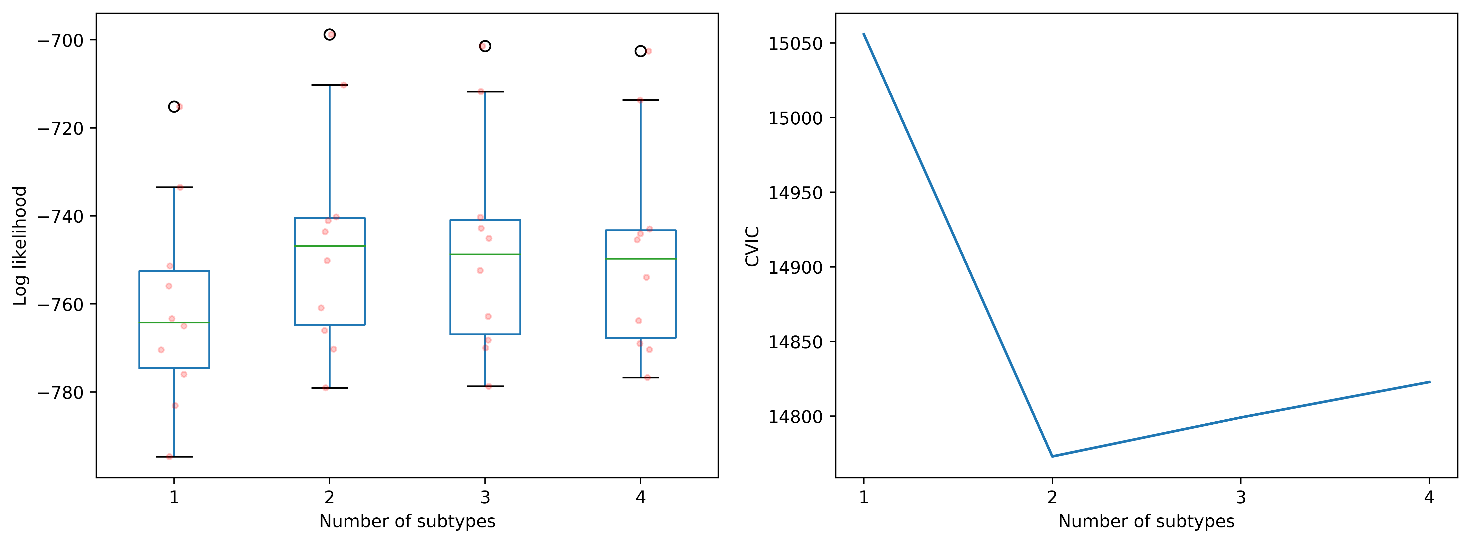


**Supplementary Figure 2. Reproducibility of subtypes under cross-validation.** The boxplots summarize the distribution of the similarity (Bhattacharyya coefficient) between subtypes estimated from each pair of cross-validation folds.

**
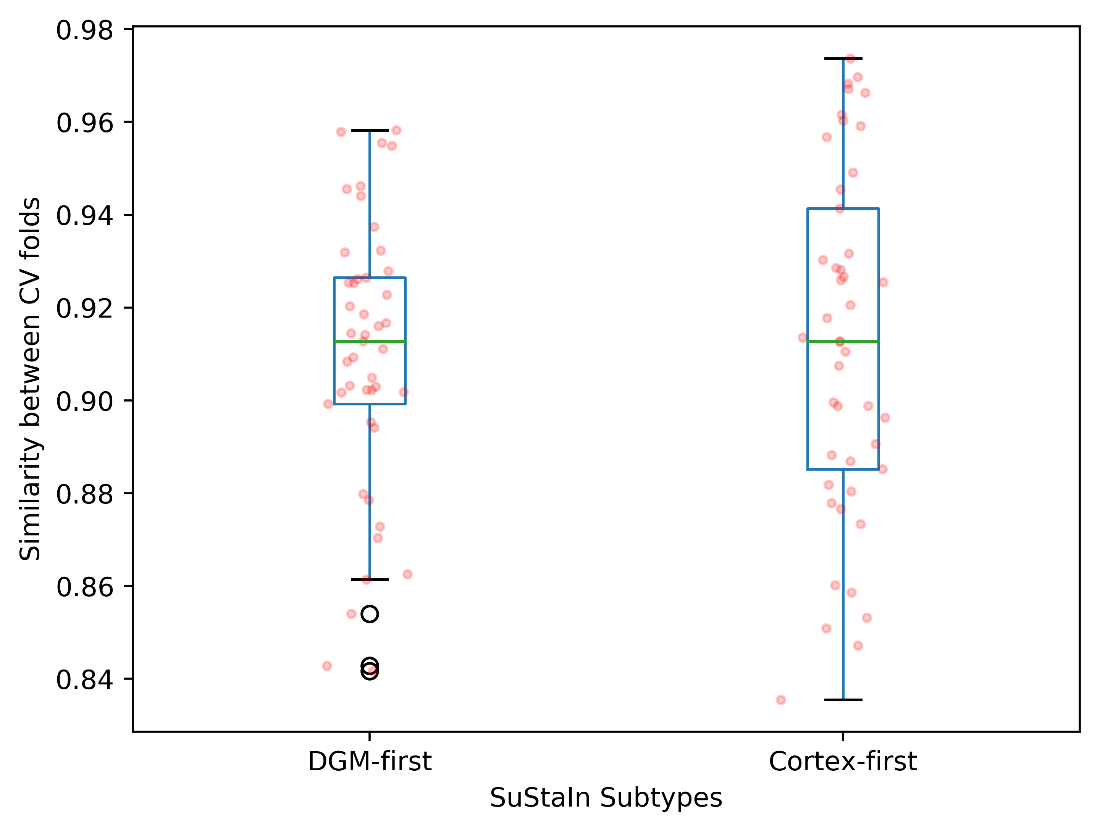
**

**Supplementary Figure 3. Relationship between SuStain classification and baseline EDSS.** Grouped scatterplot showing the association between SuStaIn stage and EDSS at baseline for both the DGM-first and Cortex-first subtypes.


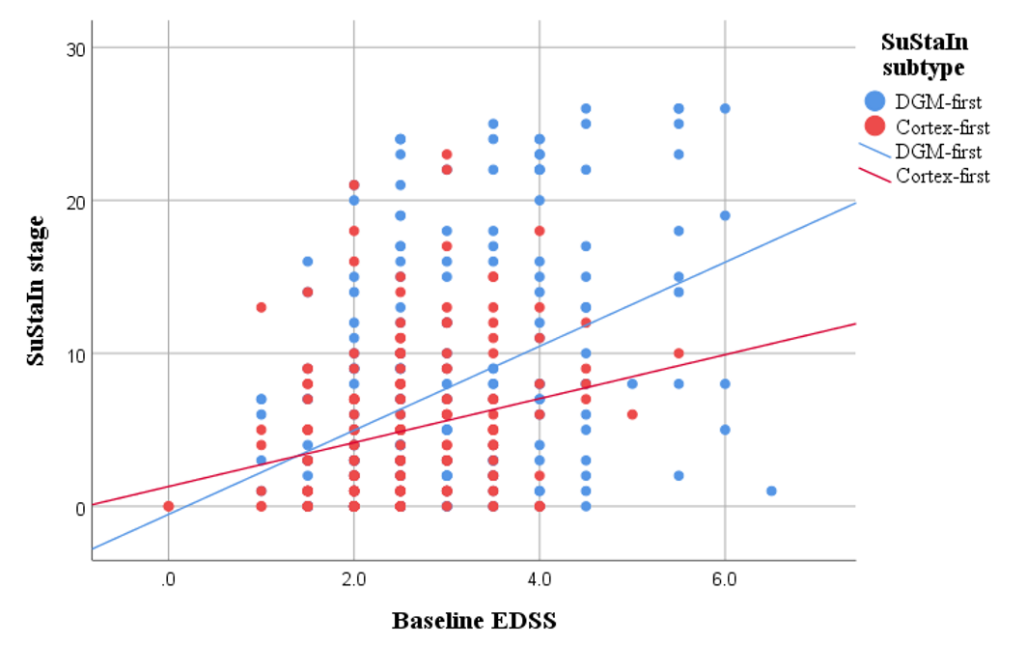


**Supplementary Figure 4. Relationship between SuStain classification and long-term disability.** Grouped boxplot showing the distribution of SuStaIn stages for each level of long-term (10 years) disability (ranging from 0 to 3 according to ambulation benchmarks, corresponding to EDSS scores <4.0, ≥4.0 and <6.0, ≥6.0 and <7.0, ≥7.0), separately for the two SuStaIn subtypes.


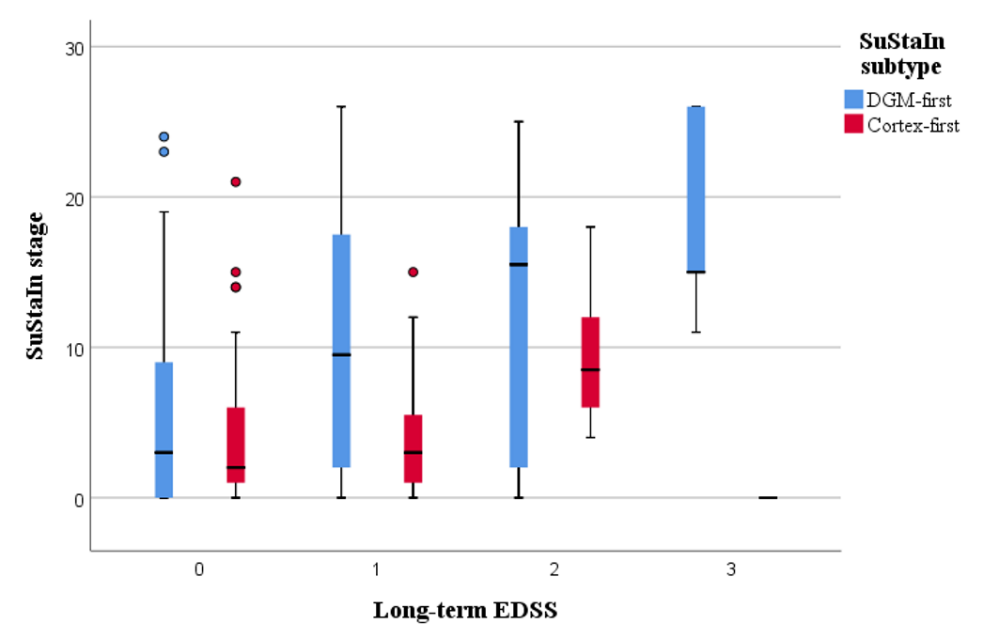


**Supplementary Figure 5. Relationship between SuStain classification and long-term cognitive impairment.** Grouped boxplot showing the distribution of SuStaIn stages for each level of long-term (10 years) cognitive impairment (ranging from 0 to 3 and corresponding to the number of impaired tests at the BICAMS battery), separately for the two SuStaIn subtypes.


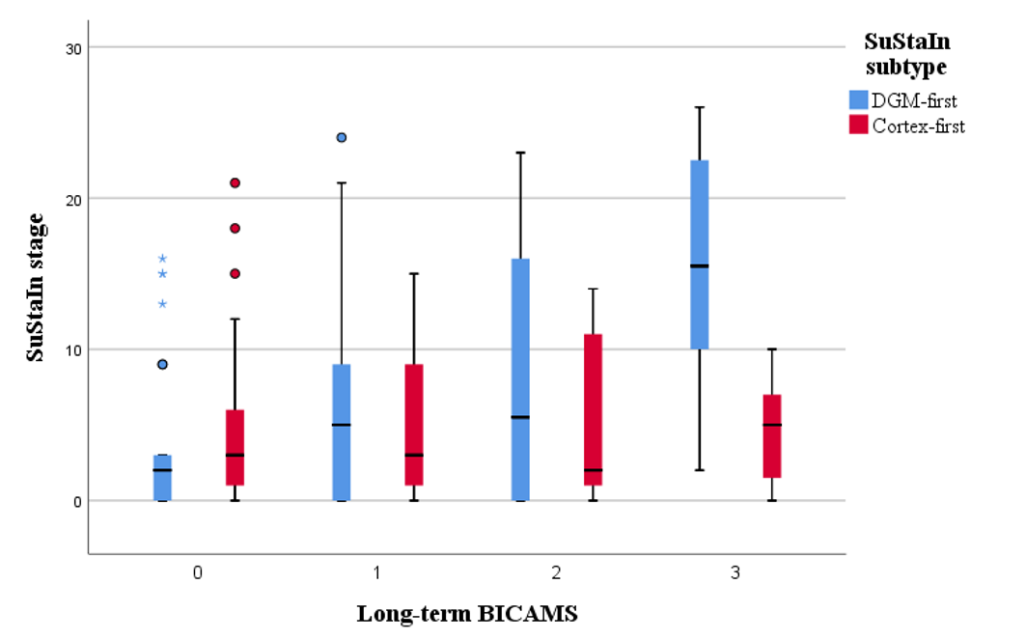


**Supplementary Figure 6. Relationship between SuStain classification and long-term clinical phenotype.** Grouped boxplot showing the distribution of SuStaIn stages for patients that retained the relapsing-remitting clinical phenotype and those who had transitioned to secondary-progressive (SP) course at the long-term (10 years) follow-up, separately for the two SuStaIn subtypes.

**
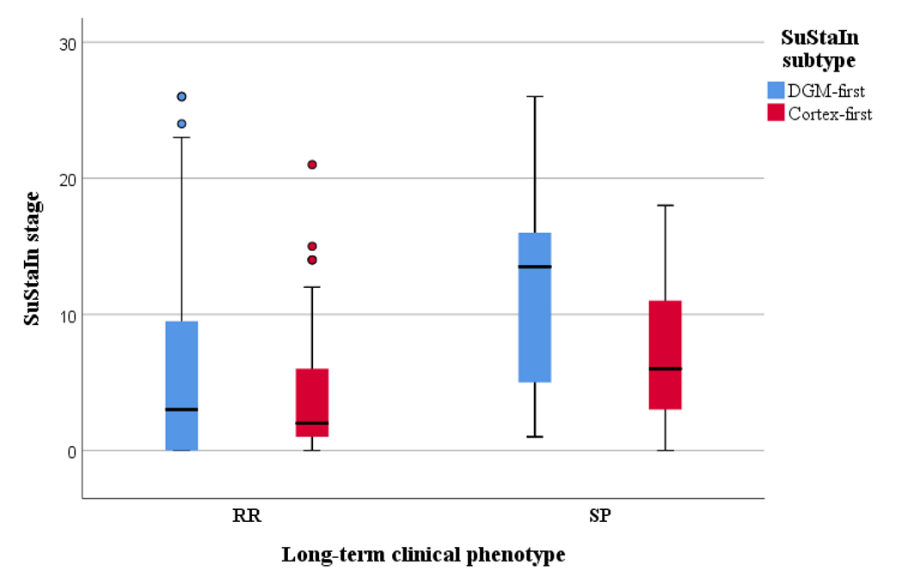
**

**References**

1 Eshaghi A, Young AL, Wijeratne PA et al (2021) Identifying multiple sclerosis subtypes using unsupervised machine learning and MRI data. Nat Commun 12:2078

2 Schmidt P (2017) Bayesian inference for structured additive regression models for large-scale problems with applications to medical imaging,

3 Tzourio-Mazoyer N, Landeau B, Papathanassiou D et al (2002) Automated anatomical labeling of activations in SPM using a macroscopic anatomical parcellation of the MNI MRI single-subject brain. Neuroimage 15:273-289

4 Young AL, Marinescu RV, Oxtoby NP et al (2018) Uncovering the heterogeneity and temporal complexity of neurodegenerative diseases with Subtype and Stage Inference. Nat Commun 9:4273
